# Vitamin Status and the Development of Postoperative Cognitive Decline in Elderly Surgical Oncologic Patients

**DOI:** 10.1245/s10434-017-6118-6

**Published:** 2017-10-20

**Authors:** Linda B. M. Weerink, Barbara L. van Leeuwen, Sofie A. M. Gernaat, Anthony R. Absalom, Monique G. Huisman, Hanneke van der Wal- Huisman, Gerbrand J. Izaks, Geertruida H. de Bock

**Affiliations:** 1 Department of Surgery, University of Groningen, University Medical Center Groningen, Groningen, The Netherlands; 2 Department of Anesthesiology, University of Groningen, University Medical Center Groningen, Groningen, The Netherlands; 3 Department of Epidemiology, University of Groningen, University Medical Center Groningen, Groningen, The Netherlands

## Abstract

**Background:**

This study aimed to evaluate the influence that serum levels of vitamin B12, folate, and homocysteine have on the development of short-term postoperative cognitive decline in the elderly surgical oncology patient.

**Methods:**

This study was part of a prospective cohort study focused on postoperative cognitive outcomes for patients 65 years of age or older undergoing surgery for a solid malignancy. Postoperative cognitive decline was defined as the change in the combined results of the Ruff Figural Fluency Test and the Trail-Making Test Parts A and B. Patients with the highest change in scores 2 weeks postoperatively compared with baseline were considered to be patients with cognitive decline. Patients with the lowest change were considered to be patients without cognitive decline. To analyze the effect of vitamin levels on the changes in postoperative cognitive scores, uni- and multivariate logistic regression analysis were performed.

**Results:**

The study enrolled 61 patients with and 59 patients without postoperative cognitive decline. Hyperhomocysteinemia was present in 14.2% of the patients. Patients with postoperative cognitive decline more often had hyperhomocysteinemia (27.9 vs 10.2%). Hyperhomocysteinemia was associated with a higher chance for the development of postoperative cognitive decline (odds ratio_adjusted_, 11.9; 95% confidence interval, 2.4–59.4). Preoperative vitamin B12 or folate deficiency were not associated with the development of postoperative cognitive decline.

**Conclusion:**

Preoperative hyperhomocysteinemia is associated with the development of postoperative cognitive decline. The presence of preoperative hyperhomocysteinemia could be an indicator for an increased risk of postoperative cognitive decline developing in the elderly.

Postoperative cognitive impairment, a decline in cognitive function for weeks or months after surgery, is a frequently reported complication.^1^,^2^ It is reported for 25–30% of elderly patients within the first 2 weeks and for 10–15% of patients 3 months after major surgery.^3^,^4^

In the past, survival was the main goal of cancer treatment. Currently, as survival has improved due to better and more advanced anesthetic and surgical techniques, quality of life after surgical cancer treatment is becoming an increasingly important issue, and preventing postoperative cognitive decline could contribute to improving quality of life.^1^

For patients with a cancer diagnosis, malnutrition is a common condition that can affect as many as 50–85% of patients, depending on the type of cancer.^1^,^2^ Among malnourished patients, 40% experience postoperative complications, whereas only 15% of patients without malnutrition experience postoperative complications.^5^,^6^

One aspect of malnutrition is a possible vitamin deficiency. Decreased serum levels of vitamin B12 and folate lead to an elevated level of homocysteine due to decreased conversion of homocysteine to methionine. Methionine is necessary for maintaining the integrity of the nervous system (Fig. 1).^7^,^8^

**Fig. 1:**
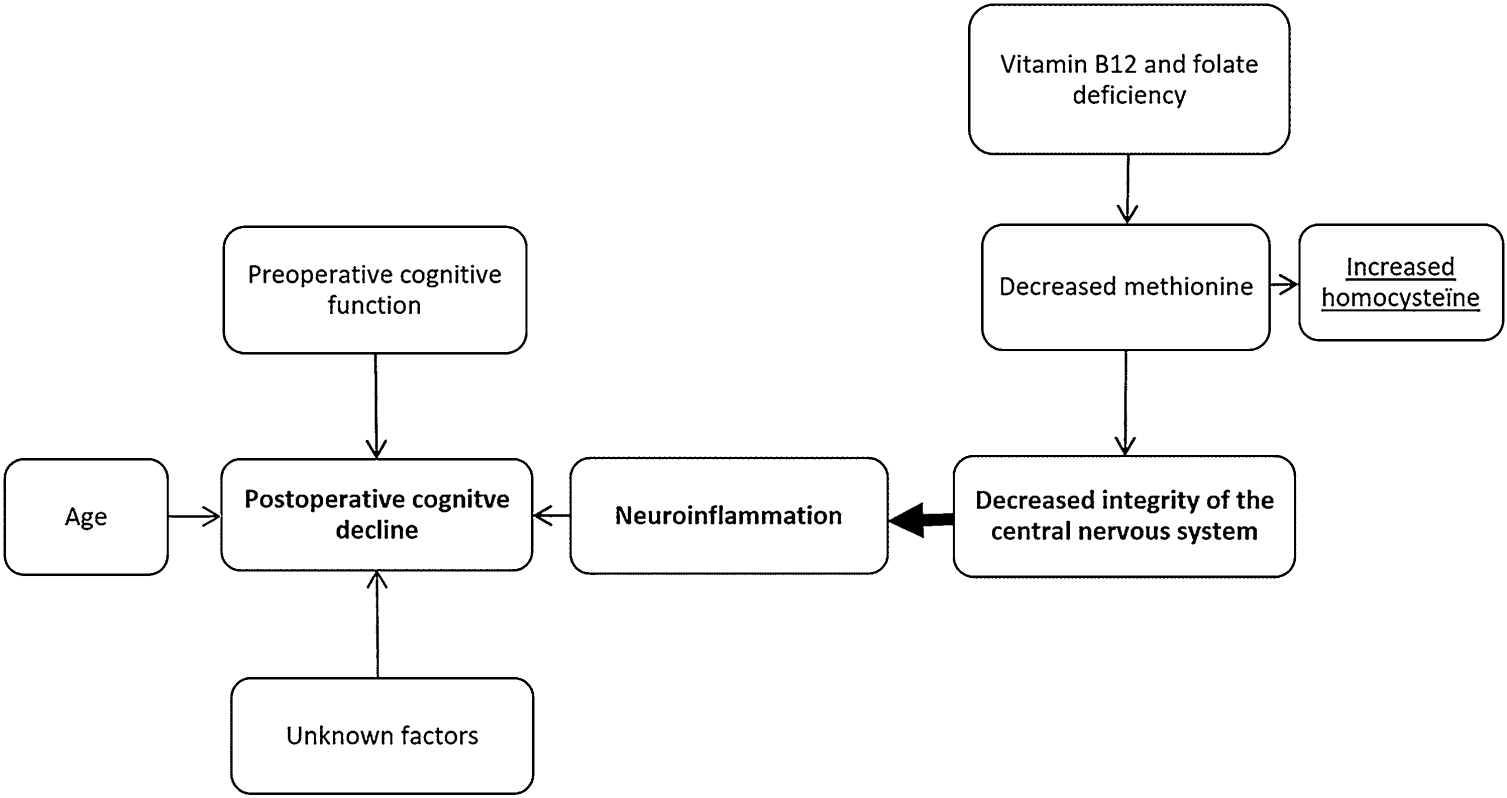
Overview of factors contributing to the development of postoperative cognitive decline

Vitamin B12 deficiency is reported to occur in 10–15% of individuals older than 60 years.^9^ Folate deficiency is prevalent in 30–35% of the elderly,^10^ and hyperhomocysteinemia is present in 29–70% of the older population.^10^–^12^ An even greater prevalence of these changes in vitamin status can be expected in elderly cancer patients due to the increased metabolic activity of the tumor, a catabolic state, or reduced food intake.^13^ These metabolic changes could contribute to an increased vulnerability of the nervous system to the inflammatory response to surgery, leading to the development of postoperative cognitive changes.

This study tested the hypothesis that patients with short-term postoperative cognitive decline more often have lower levels of serum vitamin B12 and folate and higher levels of serum homocysteine than patients without postoperative cognitive changes.

## Methods

### PICNIC Study

This study was a subanalysis of data from the ongoing prospective observational PICNIC study. This study concerning postoperative cognitive changes in elderly cancer patients started in April 2010 and aimed to assess the incidence of postoperative cognitive changes, its determinants, and its effects on quality of life. The study was conducted at the University Medical Center Groningen (Groningen, the Netherlands). The research protocol was approved by the Medical Ethical Committee of this hospital (trial no. NL45602.042.14).

Patients 65 years or age or older were enrolled in this study when they were considered eligible for surgical removal of a malignant solid tumor in the gynecologic tract, digestive tract, soft tissue/skin, or other organs under general, regional, or local anesthesia. The exclusion criteria ruled out any physical condition potentially hampering compliance with the study protocol including insufficient understanding of Dutch language, preexisting cognitive impairment, and a recent history of stroke with severe loss of cognition and function. All participating patients gave informed consent.

Preoperatively data about basic patient characteristics and medical history were obtained, and blood samples were acquired to determine biochemical parameters. Before surgery, the Groningen Frailty Index (GFI), the Mini Mental State Examination (MMSE), and the Charlson Comorbidity Index (CCI) were performed.^14^–^16^

To assess pre- and postoperative cognitive function, neuropsychological tests were conducted at baseline (before surgery) and 2 weeks postoperatively. The Ruff’s Figural Fluency Test (RFFT, parts 1–5) was used to measure strategic reasoning, planning, and ability to ignore distractors, which involved the number of unique patterns drawn during each part of the RFFT.^17^,^18^ Additionally, the number of seconds it took to complete the Trail-Making Test (TMT A and B) was used to measure information-processing speed.^19^,^20^

The preoperative tests were conducted either in the outpatient department, at the patients home during a visit, or after admission to the hospital the day before surgery. The postoperative tests were conducted 14 days after surgery, either during the admission or at the patient’s home after discharge. All tests were performed by a trained research nurse or research student. For an overview of the assessments, see Table 1.

**Table 1:** Overview of assessments in the PICNIC study

Variables	Inclusion	Before surgery (baseline)	Before surgery	2 Weeks postoperatively
Demographics				
Sex	X			
Age	X			
Body weight		X		X
Length		X		
Educational level		X		
Type of surgery		X		
Goal of surgery		X		
Cognitive tests				
Ruff figural fluency test		X		X
Trail-making test part A		X		X
Trail-making test part B		X		X
Screening instruments				
Groningen frailty index^a^	X	X		X
Mini mental state examination		X		
Charlson comorbidity index		X		
Biochemical parameters				
Serum homocysteine			X	
Serum vitamin B12			X	
Serum folate			X	

^a^At time of inclusion or before surgery

**Table 2:** Baseline characteristics of the total study population versus the patients with and without postoperative cognitive decline

Variables	Total population (*n* = 120)	Patients without postoperative cognitive decline (*n* = 59)	Patients with postoperative cognitive decline (*n* = 61)
Median age: years (range)	72.9 (65.0–89.0)	69.1 (65.0–81.0)	76.0 (66.0–89.0)
Gender: *n* (%)			
Male	59 (49.2)	30 (50.8)	29 (47.5)
Female	61 (50.8)	29 (49.2)	32 (52.5)
CCI: *n* (%)^a^			
< 4	75 (63.6)	42 (72.4)	33 (55.0)
≥ 4	43 (36.4)	16 (27.6)	27 (45.0)
GFI: *n* (%)^a^			
< 4	75 (63.0)	42 (71.2)	33 (55.0)
≥ 4	44 (37.0)	17 (28.8)	27 (45.0)
Median MMSE (range)	28.1 (20.0–30.0)	29.0 (26.0–30.0)	27.2 (20.0–30.0)
Educational level: *n* (%)^b^			
≤ Primary school	16 (13.4)	4 (6.8)	12 (20.0)
> Primary school	103 (86.6)	55 (93.2)	48 (80.0)
Vitamin statuses			
Serum vitamin B12 (range)	443.4 (57.0–1688.0)	459.0 (57.0–1688.0)	428.0 (66.0–1645.0)
Vitamin B12 deficiency: *n* (%)^c^	15 (12.5)	9 (15.3)	6 (9.8)
Serum folate: *n* (%)	0.6 (0.1–9.8)	0.4 (0.1–5.2)	0.7 (0.1–9.8)
Folate deficiency: *n* (%)^d^	107 (89.2)	55 (93.2)	52 (85.2)
Serum homocysteine (range)	10.1 (0–166.3)	7.2 (0.1–113.0)	13.1 (0.9–166.3)
Hyperhomocysteinemia: *n* (%)^e^	17 (14.2)	4 (6.8)	13 (21.7)

*CCI* Charlson comorbidity index, *GFI* Groningen frailty index, *MMSE*, mini mental state examination

^a^1 missing patient in the group patients with postoperative cognitive decline

^b^2 missing patients in the group patients with postoperative cognitive decline

^c^< 200 pg/ml

^d^< 1.0 ng/ml

^e^> 15 µmol/l

### Current Analysis

The current analysis was focused on the relationship of serum vitamin B12, homocysteine, and folate to the development of short-term postoperative cognitive changes.

Postoperative cognitive decline, as measured with the RFFT and TMT parts A and B, was defined as the standardized change over time. For each cognitive test, the raw test scores were standardized according to the preoperative standard deviation of the scores for the complete patient population of the PICNIC study. For each patient, the standardized *Z* scores for the different tests were averaged into a combined *Z* score. This was done for both the pre- and postoperative cognitive function tests. The preoperative *Z* score was subtracted from the postoperative *Z* score to create a standardized difference between the pre- and postoperative tests for each patient.

**Fig. 2:**
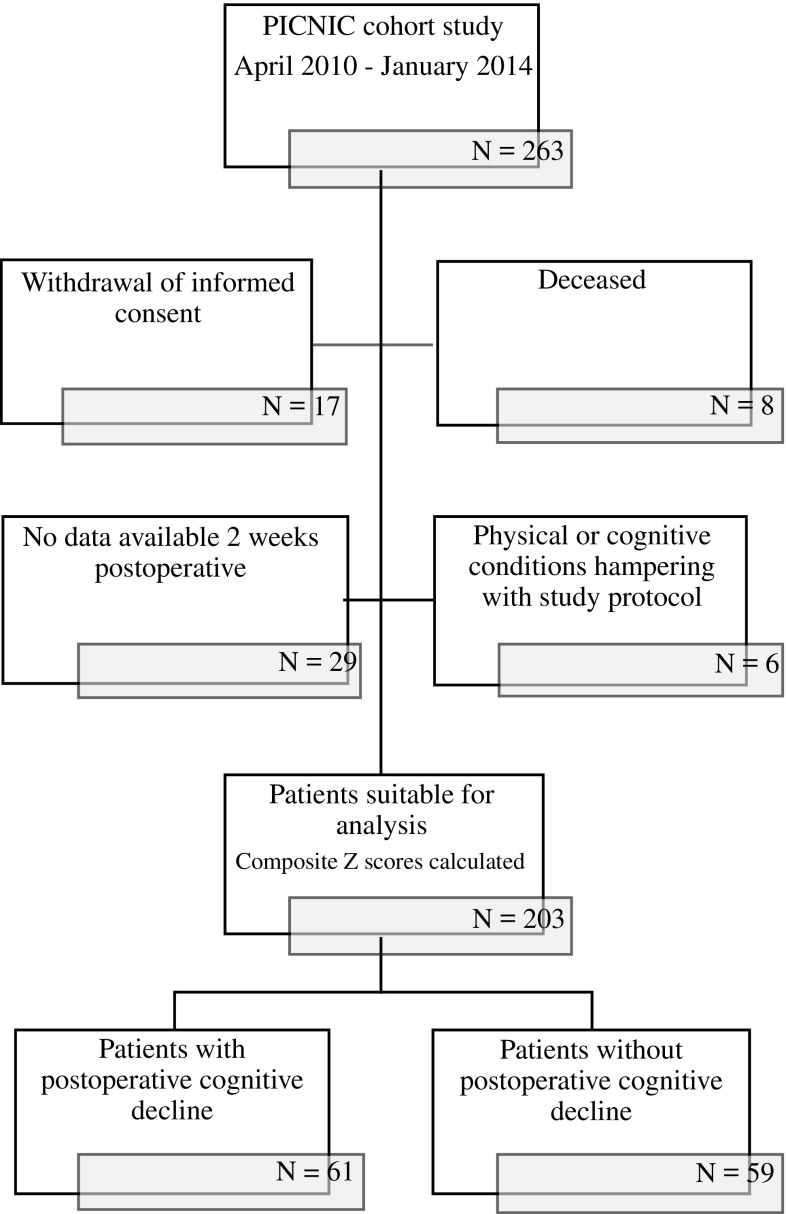
Patient selection

Patients selected from the PICNIC study cohort participated during the period between April 2010 and January 2014. The scores on the pre- and postoperative cognitive tests, RFFT and TMT parts A and part B, were used to divide the patients into two groups. From the 203 patients in the PICNIC study cohort, those with the largest positive standardized difference in scores 2 weeks postoperatively compared with the preoperative scores (i.e., the patients with the greatest decline in cognitive function) were considered to be patients with cognitive decline, whereas the patients with the largest negative standardized difference in scores were considered to be patients without cognitive decline (Fig. 2).

This analysis used the information registered in the PICNIC database. Vitamin status was assessed with serum vitamin B12, serum folate, and serum homocysteine in samples collected before surgery. Serum vitamin B12 and folate levels were analyzed using the enzyme-linked immunosorbent assay (ELISA) method (Cloud-Clone Corp, Houston, TX, USA). The standard curve had a highest concentration of 2 ng/ml. Samples were diluted 1:1 in 0.1% BSA/PBS buffer. Serum homocysteine level was analyzed with the enzyme-cycling method. Cutoff points of 200 pg/ml for vitamin B12 and 1.0 ng/ml for folate were used to define deficiency. For serum homocysteine a serum level of 15 µmol/L or higher was used to define hyperhomocysteinemia.

### Statistical Analysis

#### Power Analysis

Sample size calculation was performed on the basis of an expected 20% increase of vitamin B12 deficiency in patients with postoperative cognitive decline. A 15% vitamin B12 deficiency has been observed in elderly surgical cancer patients without postoperative cognitive changes.^9^ In elderly surgical cancer patients with postoperative cognitive decline, we expected a vitamin B12 deficiency of about 35%. Based on these differences, a sample size calculation was performed showing that a sample size of 60 elderly surgical cancer patients with postoperative cognitive decline and 60 elderly surgical cancer patients without postoperative cognitive decline achieved 80% power, given an alpha of 0.05, to show an association between vitamin B12 deficiency and postoperative cognitive decline. Based on the literature, we expected a 15% increase of folate deficiency and a 13% increase of hyperhomocysteinemia in patients with postoperative cognitive decline.^10^,^21^

#### Statistical Analyses

To describe baseline characteristics and the differences between patients with and without postoperative cognitive changes, the Chi square test was used for categorical variables, and the Mann–Whitney *U* test was used for continuous variables. For statistical analysis, the serum vitamin levels were dichotomized respectively into the presence or absence of vitamin B12, folate deficiency, and hyperhomocysteinemia. To determine the influence of serum vitamin B12, folate, and homocysteine on the development of postoperative cognitive decline, logistic regression analysis was performed for each vitamin separately, and odds ratios (ORs) with 95% confidence intervals (CIs) were estimated. To adjust for potential confounders, the baseline variables that differed significantly (*P* < 0.10) between patients with and without postoperative cognitive decline were included in the logistic regression analyses. Data analysis was performed using IBM SPSS Statistics version 22. All *P* values lower than 0.05 were considered statistically significant.

## Results

### Patients

The study enrolled 120 patients (61 with and 59 without postoperative cognitive decline). The patients with cognitive decline were significantly older than the patients without changes in postoperative cognition (Table 2). Also, the patients with cognitive decline more often had a CCI score of 4 or higher and a GFI score of 4 or higher, scored lower on the MMSE, and more frequently had an educational level of primary school or lower (Table 2). Among the 61 patients with cognitive decline, 5 (8.2%) had postoperative delirium.

The mean standardized difference between the pre- and postoperative tests for the patients with postoperative cognitive decline was 0.16, compared with –0.31 for the patients without postoperative cognitive decline.

### Vitamin Levels and Postoperative Cognitive Decline

Hyperhomocysteinemia was present in 14.2% of the patients. Vitamin B12 deficiency was present in 12.5% and folate deficiency in 89.2% of the patients. Hyperhomocysteinemia was more often present in the patients with postoperative cognitive decline (27.9%) than in the patients without postoperative cognitive changes (10.2%) (Table 3). The patients with hyperhomocysteinemia more often experienced comorbid conditions (Table 3). Vitamin B12 or folate deficiency did not differ between the patients with and without postoperative cognitive changes. The majority of the patients with and without postoperative cognitive changes had folate deficiency (Table 2).

**Table 3:** Differences in patient characteristics and comorbid conditions between patients with and without hyperhomocysteinemia

Variables	Patients with hyperhomocysteinemia (*n* = 17) *n* (%)	Patients without hyperhomocysteinemia (*n* = 103) *n* (%)
POCD	13 (76.5)	47 (46.5)
Median age: years (range)	73.6 (66.0–87.0)	72.8 (65.0–89.0)
Male gender	13 (76.5)	44 (43.6)
GFI ≥ 4	6 (35.3)	36 (36.0)
MMSE (range)	28.3 (25.0–30.0)	28.1 (20.0–30.0)
CCI ≥ 4	12 (70.6)	30 (30.3)

Comorbidities		
Cerebrovascular accident	1 (5.9)	5 (5.1)
Diabetes mellitus	6 (35.3)	10 (10.1)
Cardiac failure	6 (35.3)	12 (12.1)
Impairment of renal function	2 (11.8)	9 (9.1)

*POCD* postoperative cognitive dysfunction, *GFI* Groningen frailty index, *CCI* Charlson comorbidity index

Logistic regression analysis showed that hyperhomocysteinemia (OR_adjusted_, 11.95; 95% CI 2.41–59.35) was associated with a higher chance for the development of postoperative cognitive decline (Table 4). Vitamin B12 or folate deficiency was not associated with the development of short-term postoperative cognitive decline. The effect of the serum vitamin levels on the development of postoperative cognitive changes was adjusted by estimation for age, MMSE score, and GFI score.

**Table 4:** Factors influencing the development of postoperative cognitive decline 2 weeks postoperatively

	Unadjusted			Adjusted^a^		
	OR	95% CI	*P* Value	OR	95% CI	*P* Value
Hyperhomocysteinemia	3.73	1.14–12.24	0.030	11.95	2.41–59.35	0.002
Folate deficiency	2.38	0.69–8.20	0.170	0.25	0.05–1.43	0.120
Vitamin 12 deficiency	1.65	0.55–1.65	0.373	0.77	0.16–3.62	0.738

*OR* odds ratio, *CI* confidence interval

Logistic regression yielding ORs and 95% CIs

^a^Adjusted for age, mini mental state examination (MMSE), and Groningen frailty index (GFI)

## Discussion

Preoperative hyperhomocysteinemia, corrected for age, GFI score, and MMSE score, was associated with the development of short-term postoperative cognitive decline. Neither vitamin B12 nor folate deficiency was related to the development of postoperative cognitive changes.

In this study we focused on the effects of vitamin B12, folate, and homocysteine, and on the combined effect of each different vitamin in combination with other known risk factors on the development of postoperative cognitive decline. When those other risk factors such as age, worse performance on MMSE, and frailty were also present, an increased risk for the development of postoperative cognitive decline in patients with hyperhomocysteinemia was observed.

The development of postoperative cognitive decline is a multifactorial process. Neuro-inflammation is considered to be a possible causative factor (Fig. 1).^4^,^22^,^23^ Furthermore, age and preoperative cognitive impairment are known risk factors.^1^,^3^,^24^

In many cases, the condition remains unrecognized until patients or relatives notice difficulties related to the postoperative cognitive decline weeks or months after surgery. Although cognitive decline usually is reversible, it may be long-lasting and can have consequences with regard to quality of life.^1^,^2^ Although postoperative cognitive decline is a specific condition, distinguishing it from dementia syndromes remains difficult due to the similarity in clinical symptoms.

The current literature shows no consensus on the definition of postoperative cognitive decline and the use of specific neuropsychological tests for the diagnosis. The cognitive domains of the neuropsychological tests (strategic reasoning, planning, and the availability to ignore distractors in the RFFT, and the information processing speed for TMT parts A and B) are similar to those in other studies on postoperative cognitive decline.^1^ Practice effects for neuropsychological testing of healthy volunteers and the elderly have been described in the literature.^25^–^27^ With these practice effects taken into account, the decrease in performance on the postoperative tests becomes even more remarkable and indicates an actual decline in cognitive functioning.

In this study, postoperative cognitive decline was defined with the use of Z scores based on the method introduced by the International Study of Postoperative Cognitive Dysfunction.^3^ The patients with the largest positive standardized difference between the scores on the different cognitive tests 2 weeks postoperatively and the preoperative scores were considered to be patients with cognitive decline. With use of this method, the difference between pre- and postoperative cognitive functioning becomes apparent, and postoperative cognitive decline can be identified.

The relationship between hyperhomocysteinemia and the development of cognitive decline has been reported previously in the literature.^21^,^28^–^30^ Next to the development of cognitive decline, hyperhomocysteineima is associated with several other conditions such as stroke, atherosclerosis, ischemic heart diseases, and the development of osteoporosis in women.^31^–^34^ The effect of hyperhomocysteinemia on the specific development of postoperative cognitive changes has not been described previously.

In our study, the association between hyperhomocysteinemia and the development of postoperative cognitive decline was much stronger than the effect of hyperhomocysteinemia on cognitive changes in general, as described in the literature.^29^,^30^,^33^,^35^,^36^ The incidence of hyperhomocysteinemia in our population was lower than that reported in the literature. The causes of hyperhomocysteinemia can be primarily genetic, such as *MTHFR* 677C > T polymorphism, or acquired due to underlying diseases, medication, or lifestyle.^31^,^36^,^37^ Next to folate and vitamin B12 deficiency, renal dysfunction, unfavorable lipid profiles, increasing age and male sex are associated with hyperhomocysteinemia.^31^,^36^ Furthermore, smoking, increased coffee consumption, and probably increased alcohol consumption influences serum homocysteine levels.^31^,^32^

In our reasonably fit study population, a relatively small number of patients experienced conditions such as renal dysfunction (Table 3). The relative health of our population could be an explanation for the lower incidence of hyperhomocysteinemia. The presence of hyperhomocysteinemia indicates a diminished conversion of homocysteine to methionine.^7^,^8^ This leads to insufficient donation of methyl groups to methyl acceptors in the nervous system. Methyl is a necessary compound for maintaining the integrity of the nervous system including myelin, a number of neurotransmitters, and membrane phospholipids.^7^ Furthermore, elevated homocysteine levels may cause demyelination of cranial and peripheral nerves and of white matter in the brain.^38^ Although these effects may not be clinically manifest, it might make the brain more susceptible to further cognitive decline. The effect of neuroinflammation after surgery could possibly lead to an accelerated development of cognitive decline in an already susceptible brain. Hyperhomocysteinemia therefore serves as a marker of the vulnerable brain.

The number of patients experiencing vitamin B12 of folate deficiency is in accordance with the literature.^10^,^24^,^39^,^40^ In this study, vitamin B12 and folate deficiency were not related to the development of postoperative cognitive decline. One possible explanation is that vitamin B12 or folate deficiency separately does not influence the development of postoperative cognitive changes but that the combined effect could have an impact on the development of postoperative cognitive decline.

This study was the first to analyze the influence of serum vitamin and serum homocysteine levels on the development of postoperative cognitive changes. Current literature describing the influence of vitamin levels on cognitive decline focuses on cognitive impairment in general. Furthermore, in this study, we analyzed the effects of vitamin B12, folate, and homocysteine separately. Given the known strong association of age, preoperative frailty, and preoperative cognitive impairment with the development of postoperative decline, for each vitamin, we calculated adjusted ORs in addition to unadjusted ORs. In this way, we were able to present the independent effect of serum vitamin and hyperhomocysteinemia on postoperative cognitive decline.

In the future, consensus should be reached on the diagnostic criteria for postoperative cognitive decline based on standardized cognitive function tests. Other considerations for future research are further investigation of the relationship between hyperhomocysteinemia and the development of postoperative cognitive decline, the effect of vitamin supplementation on hyperhomocysteinemia, and eventually, the effect of nutritional interventions in patients with hyperhomocysteinemia on the development of postoperative cognitive changes.

## Conclusion

This study showed that the presence of preoperative hyperhomocysteinemia is associated with the development of mild postoperative decline in relatively healthy surgical oncology patients. The effect of preoperative hyperhomocysteinemia on the development of cognitive decline was sustained when adjusted for age, frailty, and preoperative cognitive functioning. Serum levels of vitamin B12 or folate were not associated with the development of postoperative cognitive changes.
